# Insights Into the Mechanisms Implicated in *Pinus pinaster* Resistance to Pinewood Nematode

**DOI:** 10.3389/fpls.2021.690857

**Published:** 2021-06-10

**Authors:** Inês Modesto, Lieven Sterck, Vicent Arbona, Aurelio Gómez-Cadenas, Isabel Carrasquinho, Yves Van de Peer, Célia M. Miguel

**Affiliations:** ^1^Instituto de Tecnologia Química e Biológica, Universidade Nova de Lisboa, Oeiras, Portugal; ^2^Instituto de Biologia e Tecnologia Experimental, Oeiras, Portugal; ^3^Department of Plant Biotechnology and Bioinformatics, Ghent University, Ghent, Belgium; ^4^VIB-UGent Center for Plant Systems Biology, Ghent, Belgium; ^5^Departament de Ciències Agràries i del Medi Natural, Universitat Jaume I, Castelló de la Plana, Spain; ^6^Instituto Nacional Investigaciao Agraria e Veterinaria, Oeiras, Portugal; ^7^Linking Landscape, Environment, Agriculture and Food, Instituto Superior de Agronomia, Universidade de Lisboa, Lisbon, Portugal; ^8^Department of Biochemistry, Genetics and Microbiology, University of Pretoria, Pretoria, South Africa; ^9^Biosystems and Integrative Sciences Institute, Faculdade de Ciências, Universidade de Lisboa, Lisbon, Portugal

**Keywords:** cell wall lignification, jasmonate, maritime pine, pine wilt disease, resistance genes, secondary metabolism, transcriptome, *Bursaphelenchus xylophilus*

## Abstract

Pine wilt disease (PWD), caused by the plant–parasitic nematode *Bursaphelenchus xylophilus*, has become a severe environmental problem in the Iberian Peninsula with devastating effects in *Pinus pinaster* forests. Despite the high levels of this species' susceptibility, previous studies reported heritable resistance in *P. pinaster* trees. Understanding the basis of this resistance can be of extreme relevance for future programs aiming at reducing the disease impact on *P. pinaster* forests. In this study, we highlighted the mechanisms possibly involved in *P. pinaster* resistance to PWD, by comparing the transcriptional changes between resistant and susceptible plants after infection. Our analysis revealed a higher number of differentially expressed genes (DEGs) in resistant plants (1,916) when compared with susceptible plants (1,226). Resistance to PWN is mediated by the induction of the jasmonic acid (JA) defense pathway, secondary metabolism pathways, lignin synthesis, oxidative stress response genes, and resistance genes. Quantification of the acetyl bromide-soluble lignin confirmed a significant increase of cell wall lignification of stem tissues around the inoculation zone in resistant plants. In addition to less lignified cell walls, susceptibility to the pine wood nematode seems associated with the activation of the salicylic acid (SA) defense pathway at 72 hpi, as revealed by the higher SA levels in the tissues of susceptible plants. Cell wall reinforcement and hormone signaling mechanisms seem therefore essential for a resistance response.

## Introduction

Pine wilt disease (PWD) is caused by *Bursaphelenchus xylophilus*, or pinewood nematode (PWN), which is transmitted by the insect vector *Monochamus* spp. while feeding on healthy trees. Upon entering the tree stem, PWN spreads through the resin canals, feeds on plant cells or fungi that populate the decaying tree, and breeds (Evans et al., 1996; Vicente et al., 2012; Kim et al., 2020).

During the last century, PWD has become a worldwide threat to conifer forests (Webster and Mota, 2008), being particularly damaging for trees of the genus *Pinus*. In the Iberian Peninsula, it was first detected in the late 1990's (Mota et al., 1999), spreading rapidly through Portugal and reaching Spain. In this region, it infects mostly *Pinus pinaster* trees, which are highly susceptible (Evans et al., 1996). Given the high economic and ecological value of *P. pinaster* in southwestern Europe due to its use in paper, wood, and resin production, its importance for soil protection, and as wildlife habitat, PWD has a huge impact on the local economy and environment (Webster and Mota, 2008; Vicente et al., 2012).

Remarkably, varieties with high resistance levels have been described in susceptible pine species (Toda and Kurinobu, 2002; Xu et al., 2012). In *P. pinaster*, different levels of resistance were also observed in plants after artificially inoculated with PWN (Menéndez-Gutiérrez et al., 2017a,b; Carrasquinho et al., 2018). Since control measures implemented so far have failed in stopping PWD spreading, breeding resistant varieties may be a highly effective control strategy. Breeding programs have been successfully implemented for *Pinus thunbergii, Pinus densiflora*, and *Pinus massoniana* (Toda and Kurinobu, 2002; Xu et al., 2012). For *P. pinaster*, genetic variation in susceptibility to PWN inoculation was observed in two independent studies (Menéndez-Gutiérrez et al., 2017a; Carrasquinho et al., 2018), in which a moderate family heritability for survival (0.37; Carrasquinho et al., 2018) and mortality (0.59; Menéndez-Gutiérrez et al., 2017a) after inoculation was detected, suggesting that implementation of breeding programs can be valuable. Furthermore, plants without symptoms had very few PWNs when compared to symptomatic plants (Menéndez-Gutiérrez et al., 2017a), suggesting asymptomatic plants were able to control the multiplication of PWN, showing, therefore, true resistance to the parasite (Trudgill, 1991; Woodcock et al., 2018).

The identification of the mechanisms involved in resistance to PWN may inform on effective strategies to fight the disease. In general, plant defense response initiates upon recognition of the pathogen at the cellular level (Couto and Zipfel, 2016). Cell membrane receptor-like kinases (RLKs) or receptor-like proteins (RLPs) recognize pathogen-associated molecular patterns (PAMPs) or damage-associated molecular patterns (DAMPs), initiating a series of signaling events that culminate in transcriptional reprogramming and expression of defense response genes. This pattern-triggered immunity (PTI) represents the first level of plant defense against pathogens (Dodds and Rathjen, 2010; Couto and Zipfel, 2016). However, adapted pathogens release effectors to suppress host immunity. In turn, these effectors may be recognized by intracellular nucleotide-binding/leucine-rich-repeat (NLR) receptors, inducing a more robust defense response, the effector-triggered immunity (ETI) (Cui et al., 2015). Several RLK/RLP and NLR receptors have been implicated in resistance to plant–parasitic nematodes (Sato et al., 2019; Zheng et al., 2021). However, these studies focus on sedentary and biotrophic species, and intracellular NLR receptors may not have a relevant role in resistance to migratory non-biotrophic nematodes such as PWN. The activation of PTI and ETI triggers hormone-dependent plant immune responses, such as salicylic acid (SA), jasmonic acid (JA), and ethylene (ET) pathways (Tsuda and Katagiri, 2010; Buscaill and Rivas, 2014). Other hormones like gibberellins, auxins, cytokinins, and abscisic acid (ABA), although usually associated with development or response to abiotic stresses, have also been shown to play an important role in plant–microbe interactions (De Vleesschauwer et al., 2014).

Although limited knowledge is available about the possible mechanisms involved in resistance to PWD in individuals within susceptible pine species, a few studies have focused on the comparison of transcriptional responses between PWD resistant and susceptible plants. For *P. thunbergii* (Nose and Shiraishi, 2011; Hirao et al., 2012) and *P. massoniana* (Liu et al., 2017) resistance was associated with higher expression levels of genes related to the synthesis of secondary metabolites, namely flavonoids (Kuroda et al., 2011) and terpenes (Liu et al., 2017), cell wall reinforcement, including genes related to plant cell wall lignification (Hirao et al., 2012; Liu et al., 2017), and ROS detoxification. Furthermore, higher lignification seemed to limit PWN migration in resistant *P. thunbergii* plants (Kusumoto et al., 2014). Recently, susceptibility in *P. pinaster* was associated with the activation of SA and JA pathways, as part of an inefficient trigger of the hypersensitive response (Rodrigues et al., 2021).

The first transcriptomic analysis addressing the PWN response in *P. pinaster* was based on the comparison to *P. pinea*, described as less susceptible than *P. pinaster* (Santos et al., 2012), while more recent reports in *P. pinaster* described the transcriptional changes after PWN infection during a susceptible interaction (Gaspar et al., 2017, 2020). However, despite the identification of *P. pinaster* genotypes considered resistant (Menéndez-Gutiérrez et al., 2017a,b; Carrasquinho et al., 2018), the transcriptional response associated with resistance has not been previously analyzed.

Our aim was to identify the molecular mechanisms involved in *P. pinaster* resistance to PWD. In the absence of available *P. pinaster* clones showing either susceptibility or resistance toward the PWN, we took advantage of within family variation (Menéndez-Gutiérrez et al., 2017a; Carrasquinho et al., 2018) and used half-siblings from a single family in the transcriptomic analysis. In this way, differences in gene expression resulting from genetic variation in traits other than response to PWN were minimized. While it would be interesting to extend this analysis to other families, the strategy used here contributes to highlight the most relevant genes for the PWN response by exploring the behavior of one of the top-ranking half-sib families regarding genetic effects on survival to PWN infection, previously characterized by Carrasquinho et al. (2018). We hypothesize that differences in survival to PWN infection may be related to different transcriptional responses in the first days after inoculation, as it was observed in other *Pinus* spp. (Nose and Shiraishi, 2011; Hirao et al., 2012; Xu et al., 2012; Liu et al., 2017). In order to test this, we inoculated several plants within the selected family and analyzed the differential expression (DE) in susceptible and resistant plants at 72 h post-inoculation (72 hpi) (Nose and Shiraishi, 2011; Hirao et al., 2012; Xu et al., 2012). Through a comparative transcriptomic analysis of PWN resistant and susceptible plants, complemented with the investigation of cell wall lignification and hormone signaling, we obtained the first insights into the resistance mechanisms possibly involved and detected candidate resistance genes that can be a valuable resource for future studies.

## Materials and Methods

### PWN Inoculum

*Bursaphelenchus xylophilus* isolate Bx013.003 (Carrasquinho et al., 2018; Rodrigues et al., 2021) was obtained from an infested field tree exhibiting wilting symptoms in central Portugal (39°43′33.8^′′^N, 9°01′55.7^′′^W) and was included in the collection of INIAV's Nematology Laboratory, Oeiras, Portugal. The sequence of the ITS region is available at GenBank (NCBI) under the accession number MF611984.1. Nematodes were kept in pure culture at 25 ± 1°C on a non-sporulating *Botrytis cinerea* strain grown on autoclaved barley grains. Prior to inoculation, nematodes were allowed to grow on sterilized wood. Nematodes were separated from the culture media using the “tray” method (Whitehead and Hemming, 1965) and suspended in water in a concentration of 1,000 PWN/ml.

### Plant Inoculation, Sampling, and Symptoms Evaluation

**Figure 1:**
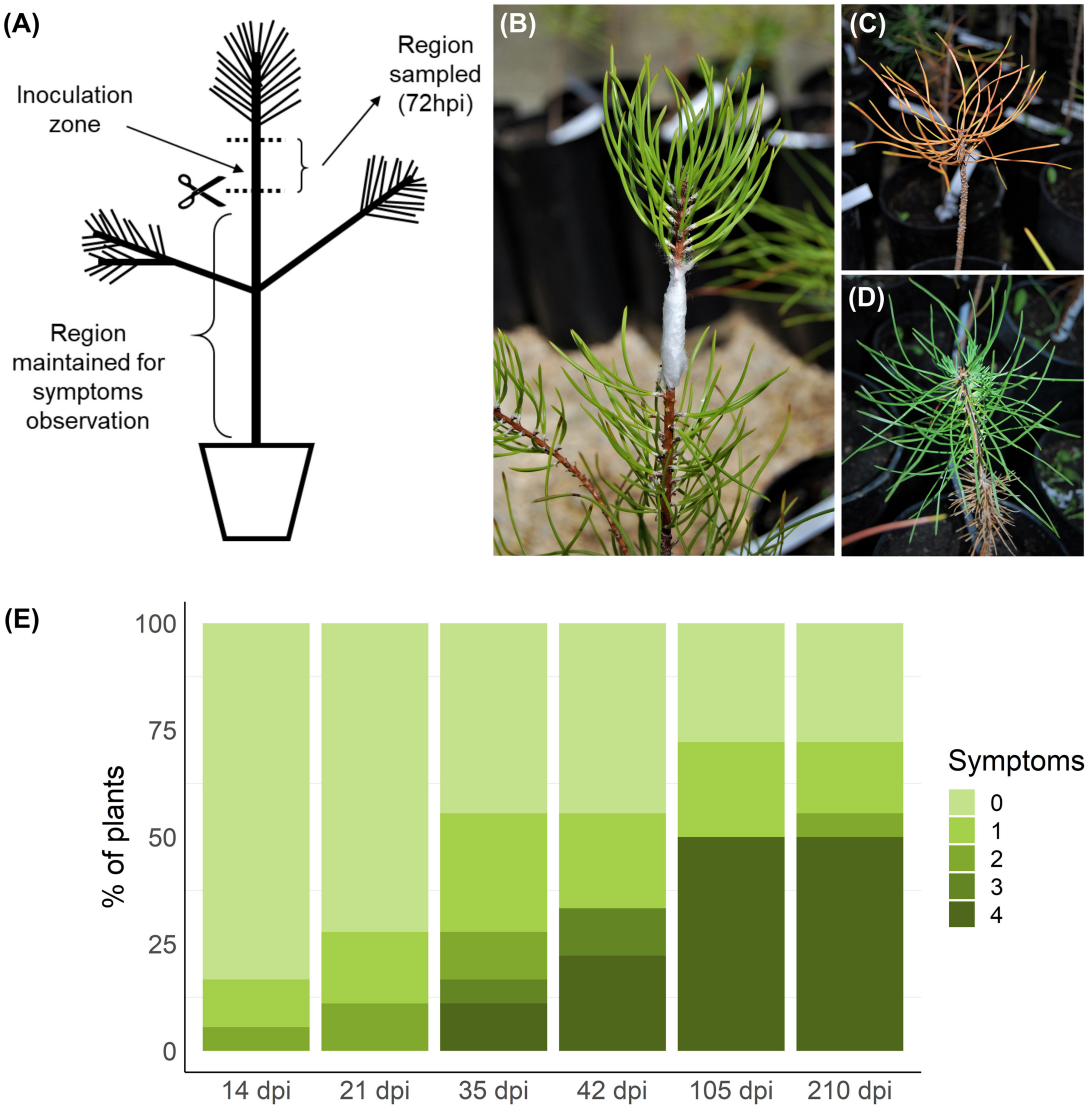
Inoculation, sampling, and symptoms observation. Plants were inoculated in the stem, below the apical region **(A,B)**. Samples of the stem, including the inoculation zone, were collected 72 h post-inoculation (hpi). After debarking, these samples were homogenized and total RNA was extracted. The remaining part of the plant, below the cutting region, was maintained for symptoms observations for 210 days post-inoculation (dpi). Symptoms were evaluated weekly and registered according to a five-level scale based on percentage of brown/wilted needles: 0, 0% **(D)**; 1, 1–25%; 2, 26–50%; 3, 51–75%; 4, 76–100% **(C)**. Symptom progression in selected timepoints is represented in **(E)**. Plants without any visible symptom at the end of the experiment were considered resistant.

Twenty-three potted 4-year-old *P. pinaster* plants from the half-sib family 440 were maintained in a greenhouse and placed according to a completely randomized experimental design. The plants were derived from seeds obtained from the mother tree 440, which is included in the reference population for PWD resistance from “Herdade da Comporta” (38°21′28.52^′′^N, 8°45′49.89^′′^W) in southern Portugal (Ribeiro et al., 2012), resulting from a mass selection program initiated in 2009. Within a half-sib family, part of the individuals may prove resistant while the majority are susceptible. Family 440 was previously characterized by Carrasquinho et al. (2018) as one of the 15 top-ranked half-sib families (among 96 evaluated families) regarding the genetic effects on survival after PWN inoculation. Predicted survival means at 157 days after inoculation ranged from 6 to 23% using 2-year-old plants, having family 440 shown a predicted survival mean of 15% (Carrasquinho et al., 2018). The plants were inoculated in September 2016, following the method of Futai and Furuno (1979). A suspension aliquot with 500 nematodes was pipetted into a small longitudinal wound made in the main stem with a sterile scalpel below the apical shoot region (Figures 1A,B). Inoculated wounds were covered with parafilm to prevent drying of the inoculum. Eighteen plants were inoculated with PWN and five controls were inoculated with sterile water. Stem samples of approximately 5 cm, including the inoculation zone (Figure 1A), were collected 72 hpi (Nose and Shiraishi, 2011; Hirao et al., 2012; Xu et al., 2013) and immediately frozen in liquid nitrogen. After removal of the inoculation zone (and apical stem), the remaining part of each plant was kept in the greenhouse and observed for symptoms weekly for a period of 210 days (Figures 1C–E). Plants were classified according to a scale from 0 (no visible symptoms) to 4 (more than 75% of needles brown/wilted) (Figure 1E). The first symptoms were visible 14 days post-inoculation (dpi) and evolved progressively until the end of the experiment. Plants presenting symptoms (1–4 in the symptoms scale) were considered susceptible, while plants without any symptoms (0) were classified as resistant. As this classification is based on external symptoms and not on nematode counting, plants here considered resistant may in fact be tolerant, maintaining a healthy phenotype despite PWN multiplication (Trudgill, 1991; Woodcock et al., 2018), although true resistance, in which plants were able to inhibit PWN multiplication, was observed in other *P. pinaster* families (Menéndez-Gutiérrez et al., 2017a). It should be noted that at 72 hpi PWNs are expected to have spread through plant tissues several centimeters away from the inoculation zone (Ichihara et al., 2000; Kusumoto et al., 2014; Son et al., 2015).

Height and diameter at the base of the stem were measured before inoculation. A two-sample unpaired *t-*test was performed using R v3.5.1 (https://www.r-project.org) to evaluate significant differences in these parameters between resistant and susceptible plants.

### Total RNA Extraction and Transcriptome Sequencing

Total RNA was extracted from each stem sample, after debarking, using the method described in Le Provost et al. (2007). RNA concentrations were measured using Qubit™ 4 Fluorometer (Thermo Fisher Scientific, Waltham, MA USA) with the RNA BR Assay Kit and integrity was verified with LabChip GX (PerkinElmer, Hopkinton, MA USA). Four susceptible and five resistant plants with the most contrasting phenotypes, i.e., plants that died faster (symptoms scale level 4) and plants without symptoms (symptoms scale level 0) during the entire observation period, were selected for library preparation, as well as four control samples. Libraries were prepared with the Illumina TruSeq Stranded mRNA Kit and sequenced on Illumina HiSeq 2500 (Fasteris, Switzerland), providing 125 bp single-end reads. Each sample was run in two independent lanes.

### Quality Control, Transcriptome Assembly, and Read Mapping

The quality of the RNA-seq data was evaluated with FastQC v0.11.2 (Andrews, 2010). Adapter and quality trimming were performed using clc_adapter_trim and clc_quality_trim, respectively, from CLC Assembly Cell v7.0.4 (Quiagen, Hilden, Germany), with default parameters.

At the moment of our analysis, a reference transcriptome was available for *P. pinaster* (Cañas et al., 2017). However, this transcriptome did not include samples submitted to any kind of biotic stress. Therefore, in order to include transcripts that may be specific to PWN infection response, we performed a *de novo* assembly with reads from all inoculated samples using Trinity v2.6.6 (Grabherr et al., 2013) with default parameters. The resulting contigs were compared with the previously available *P. pinaster* transcriptome and PWN genome (Kikuchi et al., 2011) using BLASTn (DeCypher Tera-BLASTn, TimeLogic, California, USA) and highly similar sequences (*e* ≤ 10^−5^) were filtered out. To further exclude contigs originating from PWN, a BLASTx (DeCypher Tera-BLASTx) was performed with the National Center for Biotechnology Information (NCBI) Protein database (accessed January 2019) and all the sequences with blast hits to a nematode species were excluded. In this way, 34,737 new transcripts were added to the 206,574 from the previous *P. pinaster* reference transcriptome (Supplementary Table 1). For these 34,737 transcripts, Transdecoder v2.1.0 (Haas, 2019) was used to predict protein coding regions.

Reads were mapped to the *P. pinaster* transcripts containing predicted coding regions (CDS), including both the newly predicted and the ones available in Gymno PLAZA 1.0 database (https://bioinformatics.psb.ugent.be/plaza/versions/gymno-plaza/) (70,870 transcripts). The nematode reference transcriptome (17,704 sequences) (Kikuchi et al., 2011) was obtained from WormBase ParaSite (http://parasite.wormbase.org) and used to filter out the reads corresponding to the pathogen. Reads were mapped using the BWA alignment software v0.7.5a (BWA-MEM) (Li, 2013) with default parameters. The mapping results were filtered and only uniquely mapped reads where kept for read counting using SAMtools v1.3 (Li et al., 2009). *Pinus pinaster* and PWN transcripts and respective counts were separated in two files, and only *P. pinaster* data was used for DE analysis.

### Functional Annotation

Protein sequences were obtained from Gymno PLAZA 1.0 for the available transcriptome and Transdecoder predictions were generated for the newly discovered transcripts. To functionally annotate the *P. pinaster* transcriptome, a similarity search was performed using BLASTp (DeCypher Tera-BLASTp) alignments and the NCBI RefSeq Plant database (accessed February 2019). InterProScan was used to identify protein domains, assign gene ontology (GO) terms, and assign Kyoto Encyclopedia of Genes and Genomes (KEGG) pathways. KEGG annotation was further improved by using KEGG Automatic Annotation Server (KAAS) (Moriya et al., 2007). In the set of differentially expressed genes (DEGs), transcription factors were identified and classified using iTAK (Zheng et al., 2016). Genes potentially involved in disease resistance were identified with DRAGO 2 available from the Plant Resistance Genes database (Osuna-Cruz et al., 2017).

### Differential Expression and Enrichment Analyses

The DE analysis was done using DESeq2 (Love et al., 2014) with a 0.05 false discovery rate (FDR) threshold. Results were filtered for genes with log2 fold change ≥|2|. Venn diagrams were drawn (http://bioinformatics.psb.ugent.be/webtools/Venn/).

Gene set enrichment analysis was performed using BiNGO plugin (Maere et al., 2005) for Cytoscape (Shannon et al., 2003). The hypergeometric statistical test was used, and Benjamini and Hochberg FDR was applied for multi testing correction, with a significance level ≤ 0.05. Gene ontology redundancy was decreased by using Revigo tool (Supek et al., 2011) with a soft trim threshold of 40%. Pathway enrichment analysis was performed using the hypergeometric statistical test implemented in BiNGO with the same parameters.

### Quantitative RT-qPCR Validation

To validate DE results, 10 genes with different expression patterns in susceptible and resistant plants were selected for quantitative RT-qPCR. Primers were designed using PerlPrimer (Marshall, 2004) (Supplementary Table 2). cDNA synthesis was performed from total RNA samples of three resistant, three susceptible, and three control plants using SuperScript™ IV First-Strand Synthesis System (Invitrogen, USA) and oligo(dT)_20_ primer. RT-qPCR was run in a LightCycler 480 Instrument II (Roche, Switzerland) using SYBR Green I Master (Roche) and the following conditions: 5 min at 95°C, 40 cycles of 95°C for 10 s, 58–63°C for 15 s (Supplementary Table 2), and 72°C for 12 s. Primer specificity was monitored by analyzing the melting curves. Three technical replicates were performed for each biological replicate. Transcript profiles were normalized using the reference genes *actin, 40S rRNA* (Pascual et al., 2015), and *histone H3* (de Vega-Bartol et al., 2013). Relative expression levels of candidate genes were calculated with the Pfaffl (2001) method.

### Lignin Content

Powdered stem samples were freeze-dried and 1 mg was used for determining lignin content. Acetyl bromide-soluble lignin was determined according to Foster et al. (2010) and a standard curve was generated with alkali lignin (Sigma-Aldrich, 370959). Five susceptible, five resistant, and two control samples were used for this analysis and three technical replicates were made for each biological replicate. A two-sample unpaired *t-*test was performed to evaluate significant differences between control and susceptible or resistant plants (R v3.5.1).

### Hormone Analysis

Hormone quantification was performed for five susceptible, five resistant, and four control samples. Before extraction, freeze dried powdered stem samples were weighed in 2 ml-microtubes and spiked with 25 μl of an internal standard mixture (containing ABA-d_6_, DHJA, and C^13^-SA concentration of 1 mg L^−1^) to correct for analyte loses. Extraction was carried out in 1 ml ultrapure water for 10 min in a ball mill at room temperature using 2 mm glass beads. After extraction, homogenates were centrifuged at 10,000 rpm for 10 min at 4°C and supernatants recovered. The resulting solutions were partitioned twice against an equal volume of di-ethyl ether after adjusting pH to 3.0 with a 30% acetic acid solution. The combined organic layers were evaporated under vacuum in a centrifuge concentrator (Jouan, Sant Germaine Cedex, France) and the dry residues reconstituted in 0.5 ml of a 10% aqueous methanol solution. Prior to injection, extracts were filtered through 0.20 μm PTFE syringe membrane filters and filtrates recovered in chromatography amber glass vials. Samples were analyzed by tandem LC/MS in an Acquity SDS UPLC system (Waters Corp., USA) coupled to a TQS triple quadrupole mass spectrometer (Micromass Ltd., UK) through an electrospray ionization source. Separations were carried out on a C18 column (Luna Omega Polar C18, 50 × 2.1 mm, 1.6 μm particle size, Phenomenex, USA) using a linear gradient of ultrapure acetonitrile and water, both supplemented with formic acid to a 0.1% (v/v) concentration, at a constant flow rate of 0.3 ml min^−1^. During analyses, column temperature was maintained at 40°C and samples at 10°C to slow down degradation. Plant hormones were detected in negative electrospray mode following their specific precursor-to-product ion transitions (ABA, 263>153; JA, 209>59; JA-Ile, 322>130; and SA, 137>93) and quantified using an external calibration curve with standards of known amount. To evaluate for significant differences between control and susceptible or control and resistant plants, a two-sample unpaired *t-*test was performed (R v3.5.1).

## Results

To identify genes that may be involved in resistance to PWD, an artificial PWN inoculation assay was performed with plants from a previously characterized half-sib family (Carrasquinho et al., 2018). After sampling the stem of inoculated plants at 72 hpi, plants were observed and evaluated weekly for PWD symptoms for 210 dpi. In each timepoint, plants were classified on a scale from 0 (absence of symptoms) to 4 (more than 75% of brown/wilted needles) (Figure 1E). The first symptoms were visible at 14 dpi and at 35 dpi the first plants died (level 4). After 210 dpi, 28% of the plants continued showing no symptoms (level 0) and were considered resistant. The remaining plants were considered susceptible. From the susceptible plants, 69% had died (level 4) by the end of the experiment. The first four plants reaching level 4 in the symptoms scale were selected as the susceptible plants to be sequenced by RNA-seq. Resistant and susceptible plants showed no significant differences in height and diameter at the stem base (Supplementary Figure 1).

### *De novo* Transcriptome Assembly, Functional Annotation, and Mapping

RNA-seq data from samples of stem tissue from four susceptible, five resistant, and four control plants yielded 17–20 million reads per sample, with sizes ranging between 70 and 125 bp and an average quality score of 36. The *de novo* transcriptome assembly produced 250,339 transcripts (Supplementary Table 1), from which 215,602 were highly similar to the *P. pinaster* transcriptome previously available (Cañas et al., 2017), the PWN genome (Kikuchi et al., 2011) or sequences available from other nematode species. From the remaining 34,737 transcripts, 1,445 had a predicted protein coding sequence (CDS) (Data Sheet 1). In combination with the transcripts retrieved from the Gymno PLAZA 1.0 database, a reference transcriptome of 70,870 transcripts with predicted proteins was obtained. From these transcripts, 46,625 were functionally annotated with BLASTp (DeCypher Tera-BLASTx) similarity search. Using InterProScan, at least one protein domain was identified for 44,839 transcripts, of which 31,192 had GO annotations assigned. By joining InterProScan and KAAS annotations, 17,059 transcripts were associated with at least one KEGG pathway.

Read sequences were mapped to the *P. pinaster* and PWN transcriptomes. On average, a mapping ratio of 93% was obtained, of which 69% were uniquely mapped. The percentage of reads derived from PWN in infected plants varied between samples, from 0.2 to 0.7%. PWN reads mapped to genes previously described as important for pathogenicity (Supplementary Table 3) (Kikuchi et al., 2011; Shinya et al., 2013; Espada et al., 2016), such as genes encoding enzymes involved in plant cell wall degradation (e.g., *endo-*β*-1,4-glucanase, pectate lyase, expansin*), peptidases (e.g., *cysteine proteinase, aspartic protease*), anti-oxidant proteins (e.g., *peroxiredoxin, glutathione S-transferase, thioredoxin, superoxide dismutase*) (Shinya et al., 2013), and effector protein genes, such as *venom-allergen like protein 1* (*VAP1*) and *VAP2*, which may cause the suppression of the plant immune response (Lozano-Torres et al., 2014). The expression of several pathogenicity genes during infection found in our dataset is consistent to what was described for other plant–parasitic nematodes (Haegeman et al., 2012; Goverse and Smant, 2014). For the DE analysis, only the reads uniquely mapped to *P. pinaster* transcriptome were retained.

### Differential Expression Analysis Highlighted Specific Enriched Functions and Pathways in Resistant Plants

**Figure 2:**
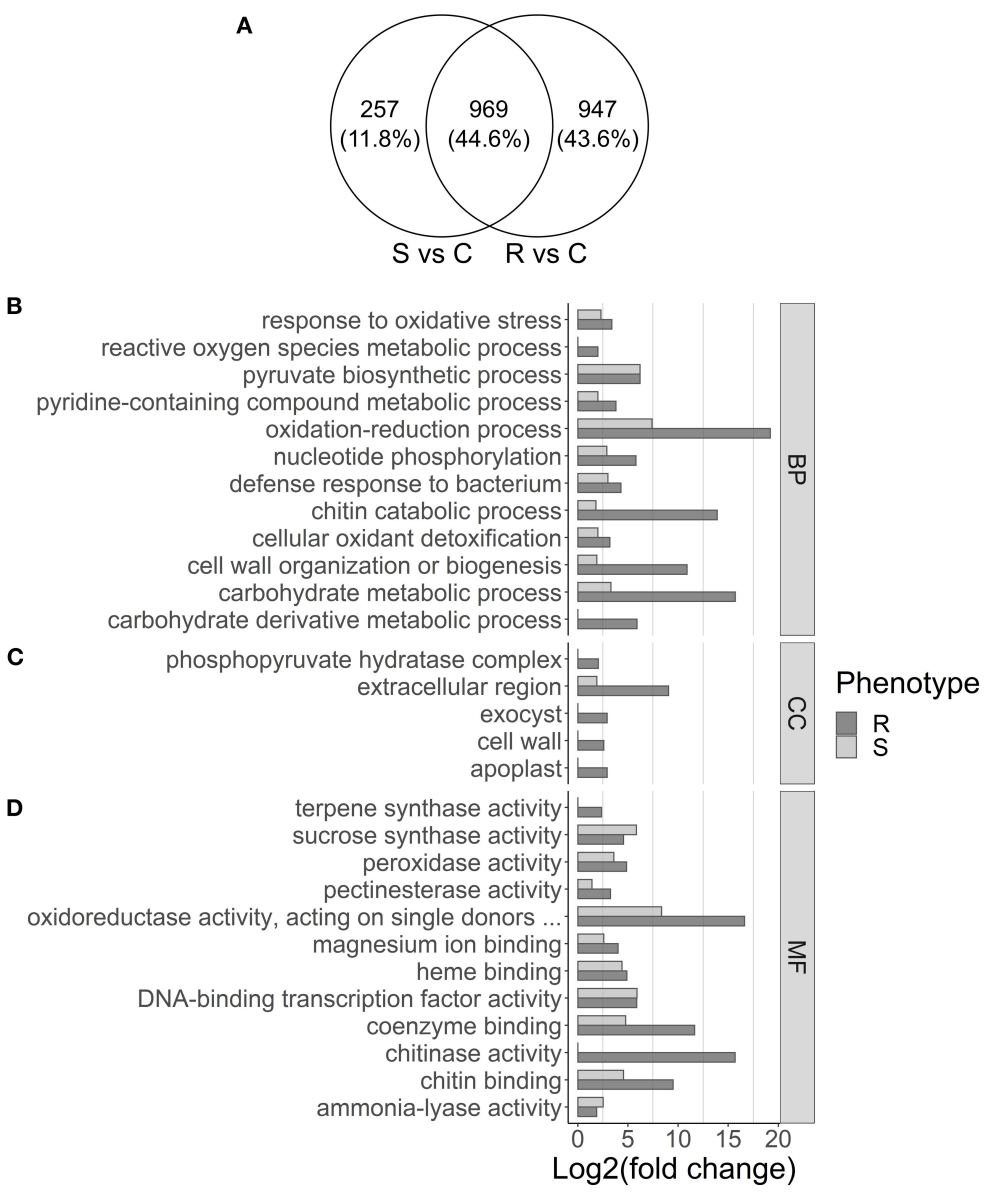
Venn diagram showing overlap of differentially expressed genes in susceptible (S) and resistant (R) samples **(A)** and gene set enrichment analysis **(B–D). (A)** Differential expression was calculated by comparing susceptible (S) or resistant (R) samples with controls (C). **(B–D)** GO terms overrepresented in the upregulated genes in resistant (dark gray) and susceptible (light gray) samples are displayed, separated by **(B)** biological process (BP), **(C)** cellular component (CC), and **(D)** molecular function (MF). The x-axis represents the significance of GO enrichment (–log10 of corrected *p*-values).

**Figure 3:**
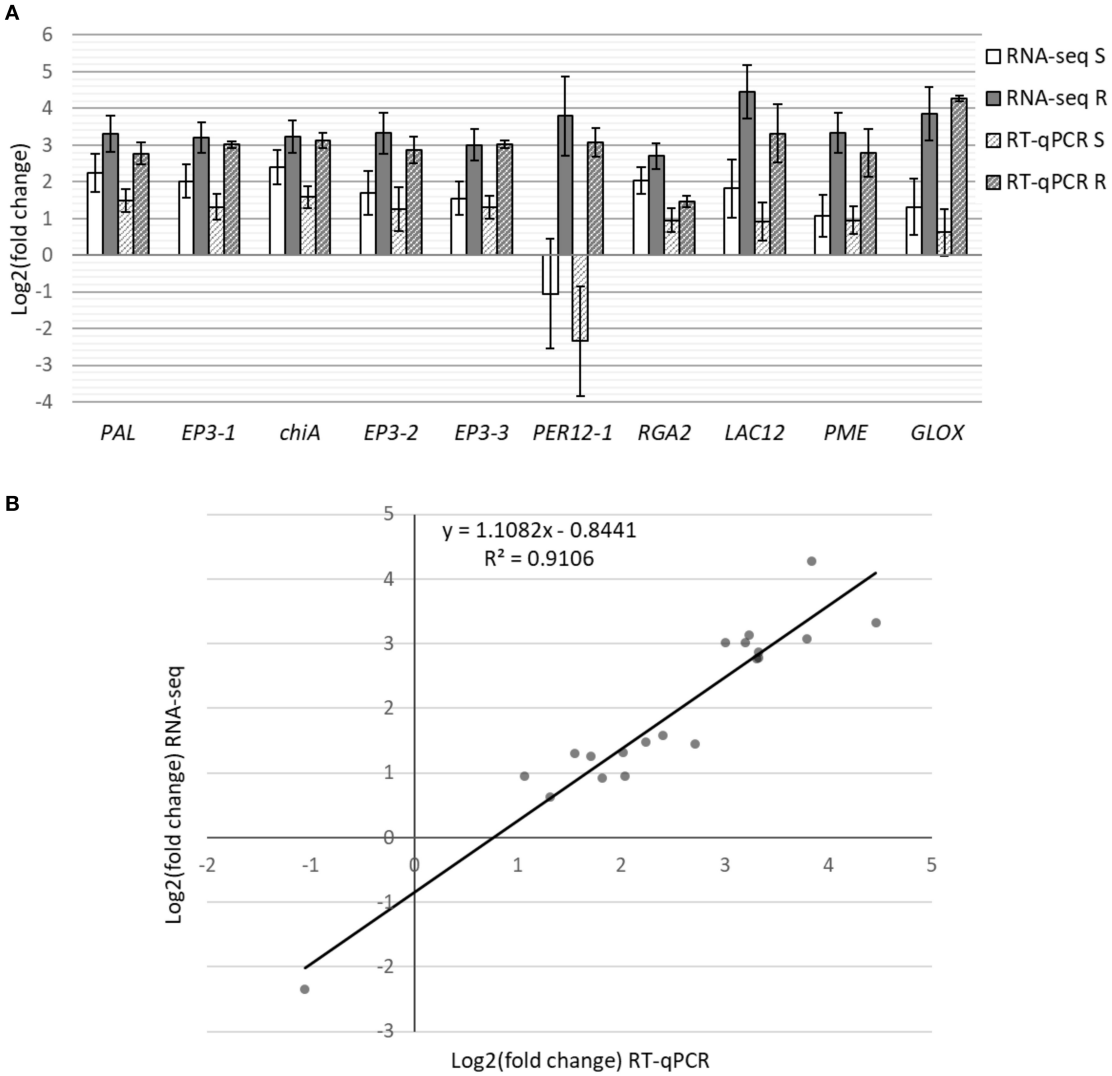
RT-qPCR analysis of 10 DEGs from the RNA-seq results. **(A)** Bars represent differential expression levels, in log2(fold change), of susceptible (white) and resistant (gray) plants in comparison with controls. Results from both the RNA-seq analysis (filled colors) and the RT-qPCR analysis (stripes) are displayed. Error bars represent the standard error of the biological replicates used for RNA-seq (4–5) and RT-qPCR (3). **(B)** Correlation of expression levels between RNA-Seq and RT-qPCR.

Differential expression analysis was performed by comparing control plants to either resistant or susceptible ones. From the 40,391 transcripts with mapped reads, 1,916 and 1,226 were differentially expressed in resistant and in susceptible plants, respectively (log2FoldChange ≥ 2, adjusted *p*-value ≤ 0.05; Supplemetary Tables 4, 5). In resistant plants, 1,182 genes were upregulated and 734 downregulated, while in susceptible plants 720 were upregulated and 506 downregulated. Part of the DEGs was shared (44.6%), while 11.8% were unique to susceptible and 43.6% were unique to resistant plants (Figure 2A). Analysis by RT-qPCR of 10 randomly selected genes show the same expression trends as the RNA-seq results (Figure 3A) with a positive correlation coefficient (*R*^2^ = 0.91, Figure 3B).

After redundancy reduction, 38 and 53 GO terms were enriched for upregulated genes in susceptible (Supplementary Table 6) and resistant plants (Supplementary Table 7), respectively. Several GO terms that are related with biotic stress response, such as DNA-binding transcription factor activity, response to oxidative stress, or defense response to bacterium, were enriched both in susceptible and resistant plants (Figures 2B–D). Gene ontology terms as the MFs chitinase activity and terpene synthase activity (Figure 2B), the BP reactive oxygen species metabolic process (Figure 2C), or the CCs cell wall and exocyst (Figure 2D), were enriched only in resistant plants.

**Figure 4:**
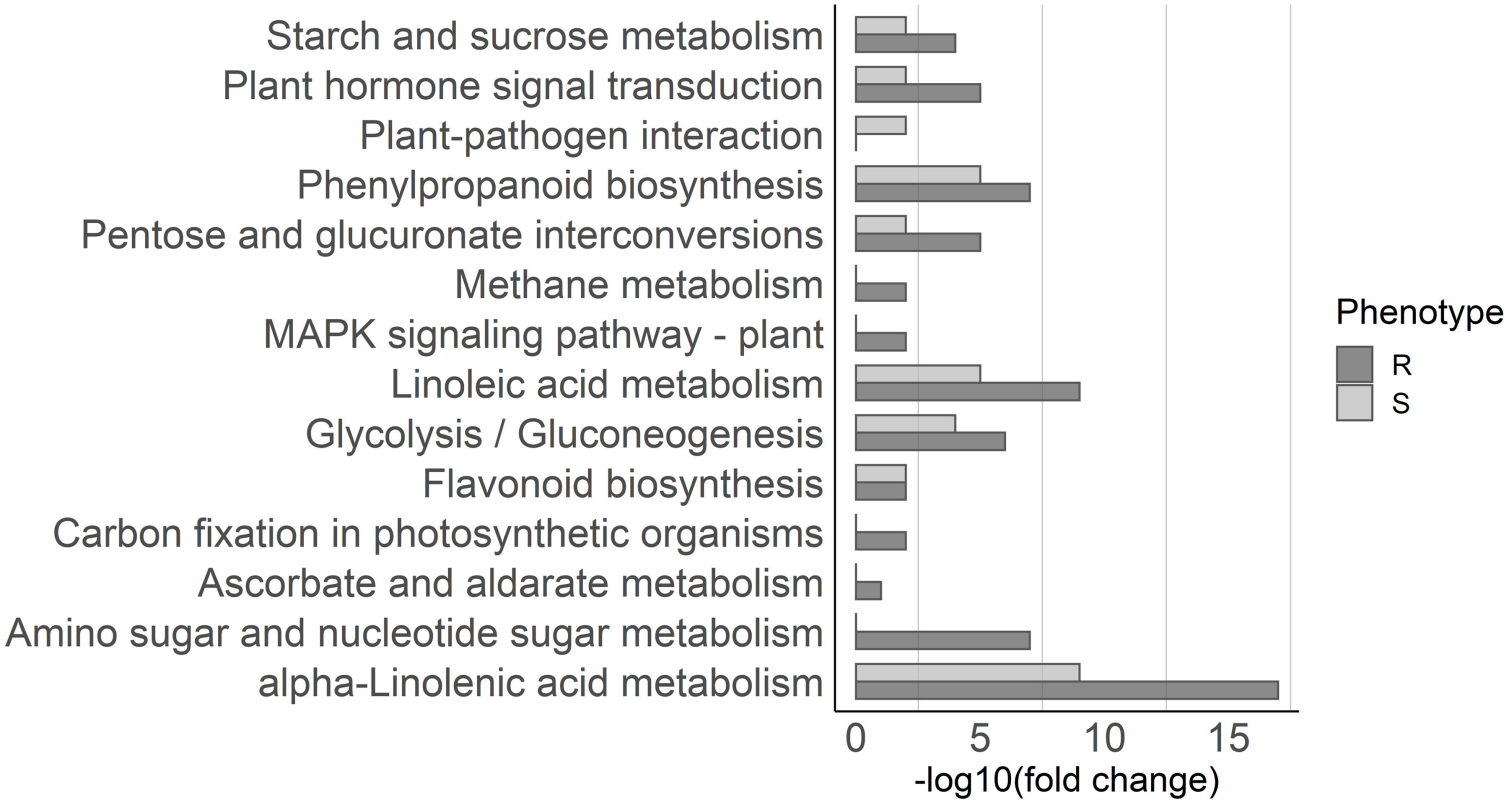
Pathway enrichment analysis. KEGG pathways overrepresented in the upregulated genes in resistant (dark gray) and susceptible (light gray) samples are depicted in the graph. The x-axis represents the significance of KEGG enrichment (–log10 of corrected *p*-values).

For the upregulated genes, 13 pathways were enriched in resistant plants and 9 in susceptible plants (Figure 4). Pathways commonly associated with biotic stress response were enriched in both resistant and susceptible plants, including alpha-Linolenic acid metabolism, which leads to the synthesis of JA, phenylpropanoid biosynthesis, which leads to the synthesis of several compounds including lignin, plant hormone signal transduction, and flavonoid biosynthesis. Pathways enriched only in resistant plants include amino sugar and nucleotide sugar metabolism and MAPK signaling pathway, while plant–pathogen interaction was enriched only in susceptible plants (Figure 4).

### Induction of Secondary Metabolism Pathways and Lignin Accumulation Was Higher in Resistant Plants

Secondary metabolites play an important role in conifers defense response and have been associated with resistance to insects and pathogens (Keeling and Bohlmann, 2006; Eyles et al., 2010; Ahuja et al., 2012). Although several genes involved in secondary metabolism pathways were differentially expressed after inoculation, a few genes related to the biosynthesis of terpenoids, such as *AS* (*bifunctional abietadiene synthase*, unigene128167), *LPS* (*bifunctional levopimaradiene synthase*, unigene10412), or *GERD* [*(-)-germacrene D synthase*, unigene144607 and unigene8510] had higher expression levels in resistant plants (Supplementary Figure 2). Likewise, a few genes from the flavonoid biosynthesis pathway were more expressed in resistant plants (Supplementary Figure 2), such as *CHS4* (*chalcone synthase 4*, isotig47436), *CHS2* (unigene147178), and *LDOX* (*leucoanthocyanidin dioxygenase*, unigene210255).

**Figure 5:**
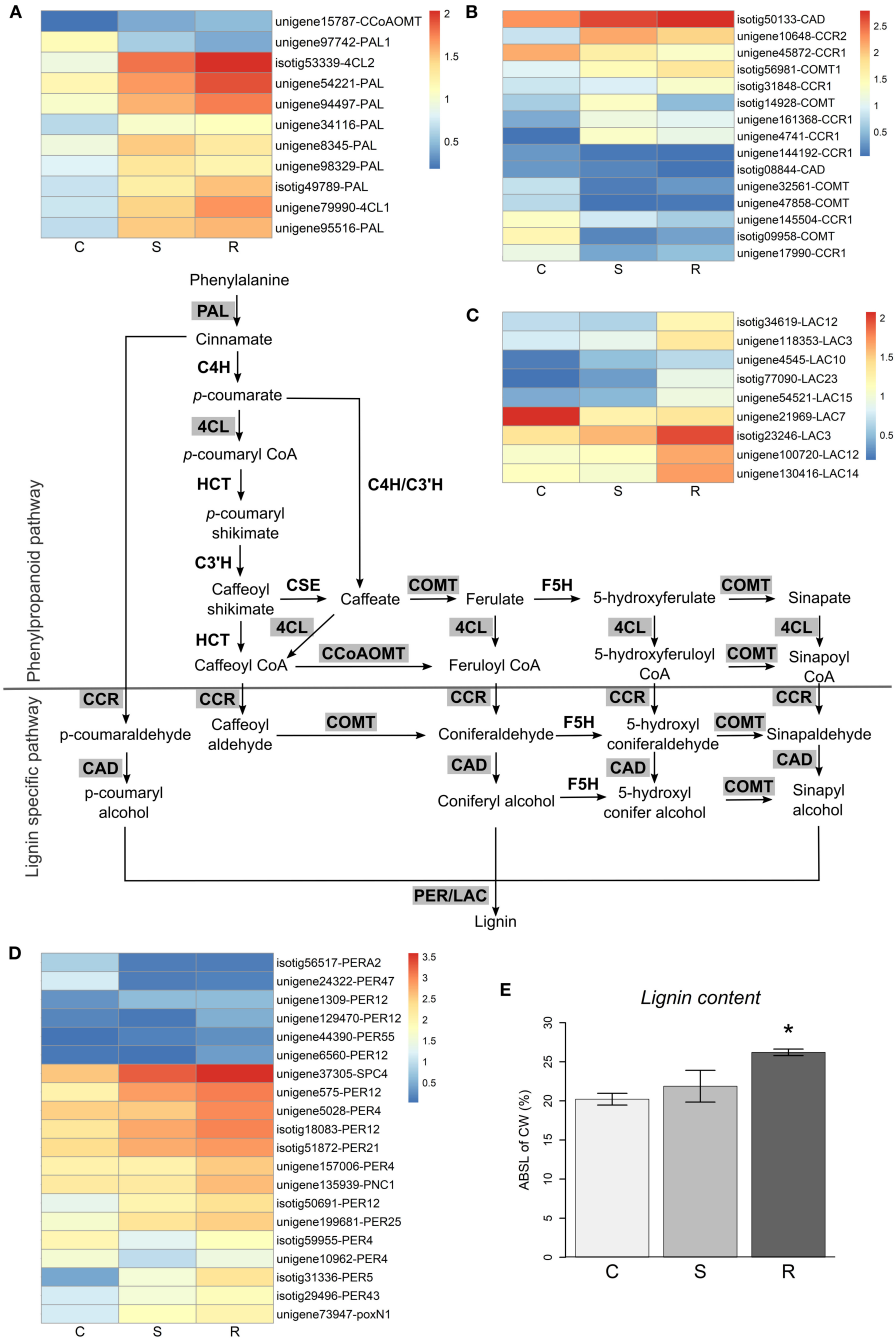
Lignin biosynthesis pathway. Lignin biosynthesis pathway is represented (adapted from Xie et al., 2018), with the differential expressed genes highlighted in gray. Heatmaps represent log10(TPM) values of differentially expressed genes in the general phenylpropanoid pathway **(A)** and the lignin specific pathway **(B–D)**. The final steps of lignin synthesis are carried out by laccases (LAC, **C**) and peroxidases (PER, **D**). The percentage of acetyl bromide soluble lignin of cell wall (ABSL of CW) measured in control (C), susceptible (S), and resistant (R) plants is represented in **(E)**. Error bars represent the standard error of the mean. Significant differences between control and inoculated plants, using Student's *t-*test, are indicated by an asterisk (*p-value < 0.05).

In contrast, a high number of genes in the phenylpropanoid biosynthesis pathway had different expression levels in resistant and susceptible plants (Figure 5). Several genes involved in lignin synthesis, including *peroxidase* (*PER*, Figure 5D) and *laccase* (*LAC*, Figure 5C) genes (Vogt, 2010; Xie et al., 2018), were more expressed in resistant plants. In addition, genes encoding for aldehyde oxidase (GLOX) enzymes, which produce hydrogen peroxide, a molecule necessary for lignin polymerization by PERs, had considerably higher expression levels in resistant plants (Supplementary Figure 3). These results, suggesting the induction of lignin biosynthesis, were supported by experimental determination of the lignin content in stem tissues, which detected a significantly higher amount of lignin in resistant plants when compared to controls, while susceptible plants were not significantly different from controls (Figure 5E). Hydrogen peroxide may also play an important role in the activation of the plant defense response and is toxic for pests and pathogens (Holbein et al., 2016). The higher production of ROS was reflected by the high number of oxidative stress response genes upregulated (Supplementary Figure 3).

### Jasmonate Response Was Induced in Inoculated Plants

**Figure 6:**
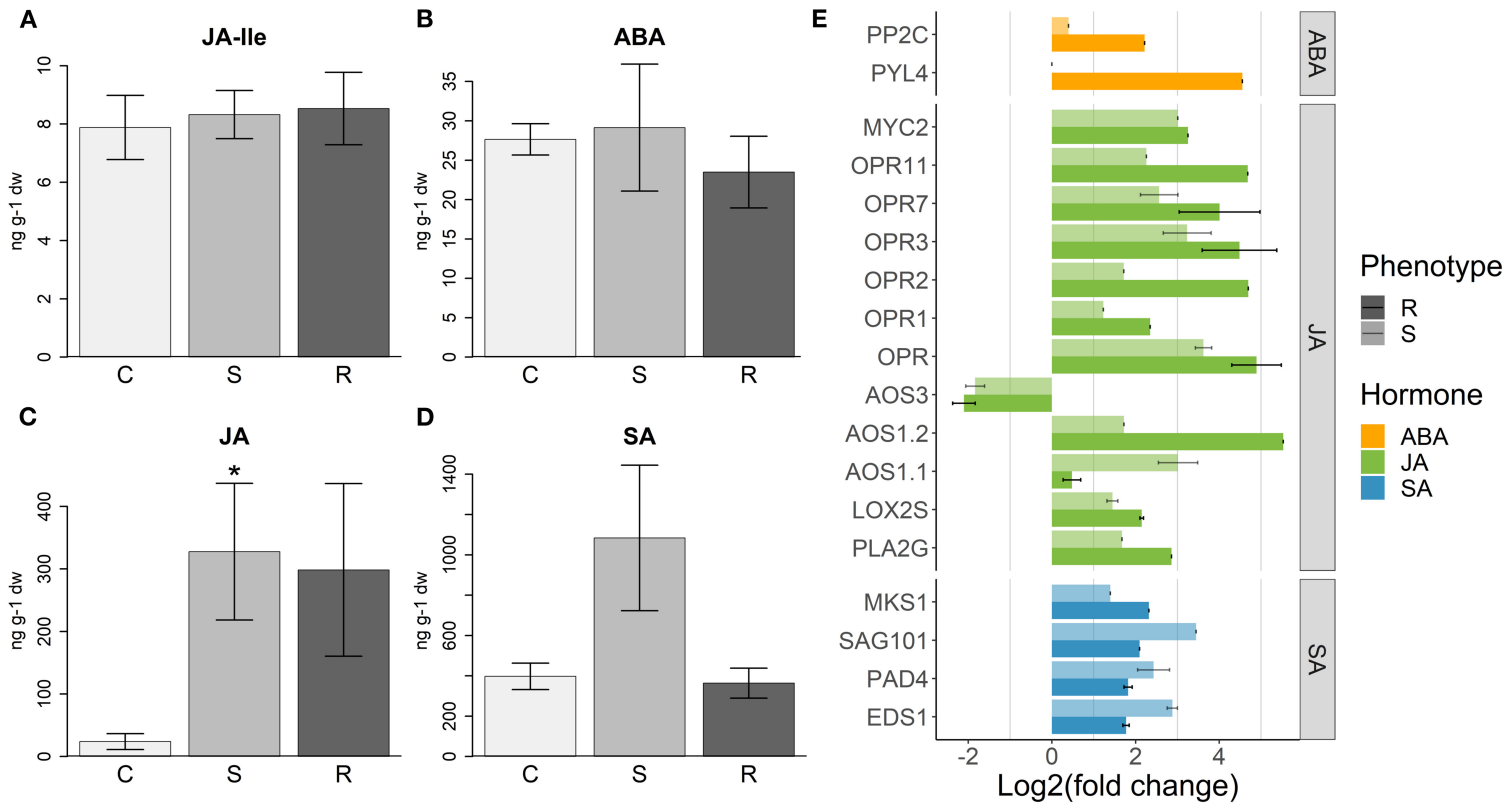
Hormone response to PWN inoculation. **(A)** Levels of jasmonate-Illenine (JA-Ile), **(B)** abscisic acid (ABA), **(C)** jasmonic acid (JA), and **(D)** salicylic acid (SA) (ng per 1 g of plant dry weight) measured in control (C), susceptible (S), and resistant (R) plants. Error bars represent the standard error of the mean. Significant differences between control and inoculated plants, using Student's *t-*test, are indicated by an asterisk (*p-value < 0.05). **(E)** Differential expression of hormone responsive genes in resistant (R) and susceptible (S) plants, compared to controls. For each gene annotation, the average of the log2(fold change) is represented. Error bars represent the standard error of the mean. For more details about the genes used and respective functional annotations see Supplementary Table 9.

Several genes involved in the JA biosynthesis pathway were upregulated in inoculated plants, with *Lipoxygenase* (*LOX*), *phospholipase A2* (*PLA2G*), and *12-oxophytodienoic acid reductase* (*OPR*) genes showing higher expression levels in resistant plants (Figure 6E). Analysis of hormone levels in the several sample types detected similar JA-Ile levels in resistant, susceptible, and control plants (Figure 6A), while the JA levels were higher only in inoculated plants (Figure 6C), although with no significant differences between resistant and susceptible plants.

**Figure 7:**
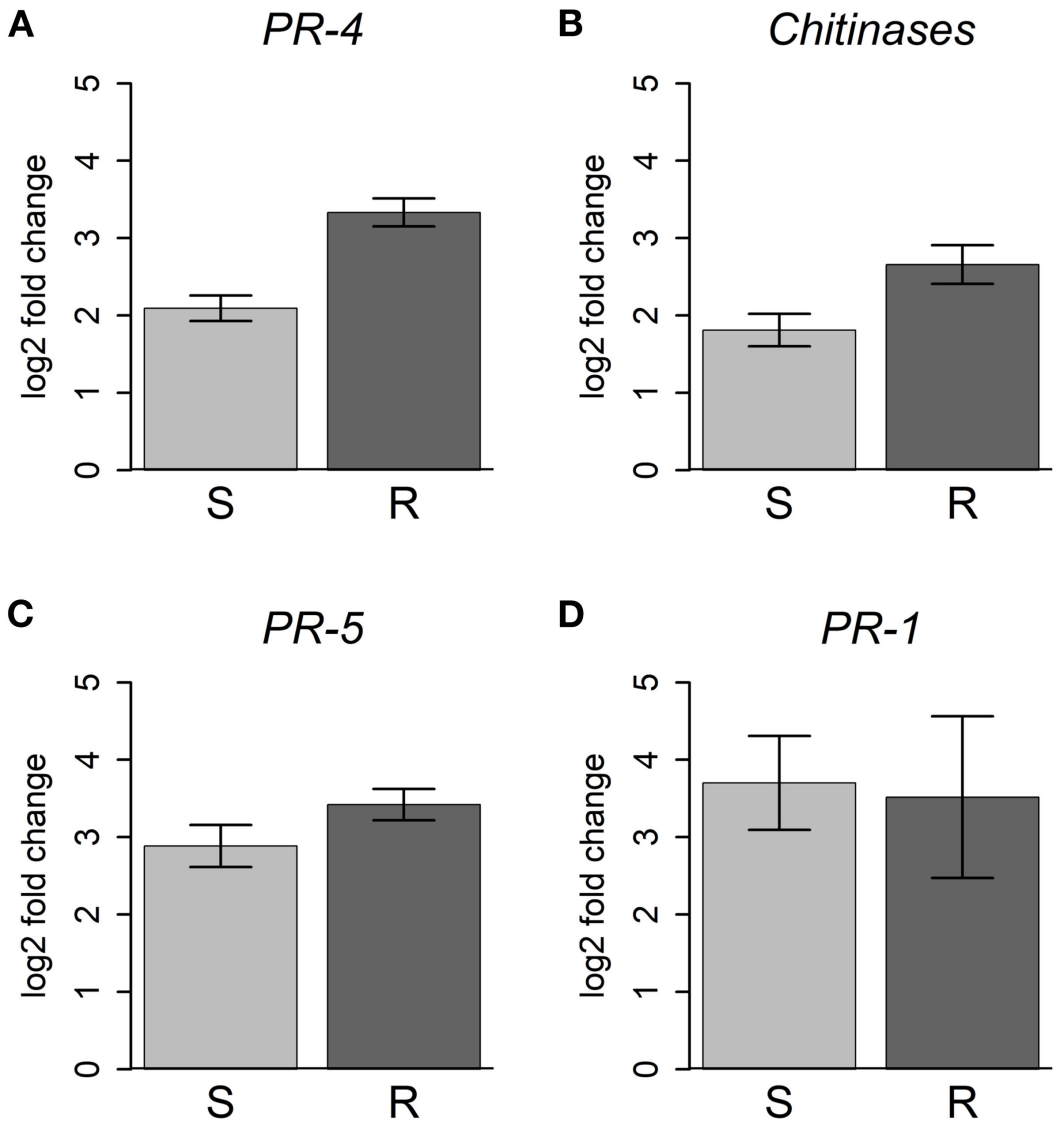
Differential expression of *PR* and *chitinase* genes. For each gene family, the average of the log2(fold change) is represented for resistant (R) and susceptible (S) plants, compared to controls. Error bars represent the standard error of the mean. **(A)** *PR-4*, 3 genes; **(B)** *chitinase*, 25 genes; **(C)** *PR-5*, 16 genes; **(D)** *PR-1*, 4 genes. For more details about the genes used and respective functional annotations see Supplementary Table 9.

Consistent with these data, JA induced transcription factors, such as *ethylene response factors* (*ERF*), *MYC2* and the negative regulators *JAZ*/*Tify* were upregulated in all inoculated plants (Supplementary Figure 4). However, 25 *chitinase*, 3 *PR-4*, and 16 *PR-5* genes, usually associated with JA response (Davis et al., 2002; Piggott et al., 2004), were more strongly upregulated in resistant plants (Figures 7A–C).

Abscisic acid may act synergistically with JA in the activation of the MYC branch of JA response (Pieterse et al., 2011). In turn, JA induces the expression of *PYL4*, which encodes for an ABA receptor (Lackman et al., 2011). In our results, we observed the upregulation of *PYL4* concomitantly with *PP2C* (Merlot et al., 2001), an ABA signaling repressor gene (Figure 6E), only in resistant plants. Five transcription factors of the NAC family, implicated in ABA–JA interactions (Pieterse et al., 2011), were upregulated in all inoculated plants, although with higher intensity in resistant ones (Supplementary Figure 4). Despite these differences in gene expression, the amount of ABA measured in the samples was similar between control and inoculated plants, with resistant plants tending to have a smaller amount (Figure 6B).

### Salicylic Acid Response Is Induced in Susceptible Plants

Genes encoding for proteins that induce the synthesis and accumulation of SA, namely *EDS1, PAD4*, and *SAG101* (Wiermer et al., 2005; Caarls et al., 2015), were more expressed in susceptible plants (Figure 6E). On the other hand, a gene encoding for the SA signaling suppressor MKS1 (Andreasson et al., 2005; Pieterse et al., 2011) was more upregulated in resistant plants (Figure 6E). In accordance with these results, the induction of the SA response in susceptible plants was validated by quantifying SA levels, which was higher in these plants than in controls and resistant plants (Figure 6D).

Several *WRKY* transcription factors, involved in the SA response pathway (Caarls et al., 2015), were upregulated in both susceptible and resistant plants, with a few showing higher expression in the susceptible ones (e.g., *unigene36207-WRKY23, isotig49008-WRKY50*) (Supplementary Figure 4). In addition, the SA responsive *pathogenesis-related protein 1* (*PR-1*) genes were also upregulated in all inoculated plants (Figure 7D). Although not significantly different, there seems to be a tendency for higher expression of *PR-1* genes in susceptible plants.

### Putative Resistance Genes Showed Different Expression Patterns in Resistant and Susceptible Plants

The analysis with DRAGO 2 identified a set of genes that encode for the characteristic domains of proteins described in the literature as having a role in resistance to pathogens (Osuna-Cruz et al., 2017), including RLK, RLP, protein kinases, and nucleotide-binding domain leucine-rich repeat (NLR) proteins (Table 1, Supplementary Table 8). The RLP differ from RLK by the presence of a kinase domain and genes in which this domain was not detected were classified as RLPs. Several RLPs and RLKs were more expressed in resistant plants (e.g., unigene148155-IRL6, DN63749_c0_g2_i1-RLK, unigene73543-PIRL3) (Table 1, Supplementary Table 8), while others were more expressed in susceptible plants (e.g., isotig67777-FLS2, unigene102513-RLP30, isotig84710-PXC2). Most genes encoding intracellular receptors NLRs had higher expression levels in resistant plants (Table 1).

**Table 1:** Putative resistance genes detected by DRAGO tool (selected).

**Transcript**	**Drago annotation**	**Blastp annotation**	**Gene**	**Log2 fold change**	
				**Sus**	**Res**
isotig36950	RLP	PREDICTED: probable disease resistance protein At4g33300 [*Nelumbo nucifera*]	*PDR*	2.73	**4.12**
DN63749_c0_g2_i1	RLP	Receptor-like serine/threonine-protein kinase At1g78530 isoform X1 [*Physcomitrella patens*]	*RLK*	0.00	**3.86**
unigene73543	RLP	Plant intracellular Ras-group-related LRR protein 3 [*Cynara cardunculus var. scolymus*]	*PIRL3*	0.49	**3.40**
DN45869_c0_g1_i1	RLP	Probable leucine-rich repeat receptor-like protein kinase At1g35710 [*Medicago truncatula*]	*RLK*	1.70	**3.11**
unigene148155	RLP	PREDICTED: plant intracellular Ras-group-related LRR protein 6 [*Erythranthe guttata*]	*IRL6*	1.14	**3.03**
unigene104083	RLP	PREDICTED: plant intracellular Ras-group-related LRR protein 6 [*Erythranthe guttata*]	*IRL6*	0.75	**2.91**
isotig19381	RLP	Probable leucine-rich repeat receptor-like protein kinase At1g35710 [*Durio zibethinus*]	*RLK*	1.24	**2.90**
isotig55894	RLP	LOW QUALITY PROTEIN: probable leucine-rich repeat receptor-like protein kinase At1g35710 [*Durio zibethinus*]	*RLK*	0.51	**2.04**
unigene10412	RLP	putative disease resistance protein RGA3 [*Aegilops tauschii subsp. tauschii*]	*RGA3*	−21.84	**0.05**
isotig82402	RLP	PREDICTED: protein SUPPRESSOR OF npr1-1, CONSTITUTIVE 1-like [*Gossypium hirsutum*]	*SNC1*	−22.04	–**0.07**
unigene75605	RLP	Leucine-rich repeat receptor-like protein kinase TDR [*Glycine max*]	*TDR*	**3.87**	2.30
DN44984_c1_g1_i1	RLP	Receptor-like protein EIX2 isoform X1 [*Glycine max*]	*EIX2*	**3.90**	2.19
DN44458_c0_g1_i2	RLP	PREDICTED: receptor-like protein kinase HSL1 [*Elaeis guineensis*]	*HSL1*	**4.63**	2.15
isotig67777	RLP	LRR receptor-like serine/threonine-protein kinase FLS2 [*Quercus suber*]	*FLS2*	**2.54**	0.07
unigene102513	RLP	PREDICTED: receptor like protein 30-like [*Vitis vinifera*]	*RLP30*	**1.81**	−2.52
isotig56462	RLP	Receptor-like protein kinase HSL1 [*Papaver somniferum*]	*HSL1*	–**0.55**	−2.17
isotig38664	RLP	Receptor-like protein 12 [*Durio zibethinus*]	*RLP12*	**0.54**	−2.19
unigene120230	RLP	Putative leucine-rich repeat receptor-like serine/threonine-protein kinase At2g24130 [*Setaria italica*]	*RLK*	**3.26**	1.28
unigene98132	RLK	PREDICTED: receptor kinase-like protein Xa21 isoform X1 [*Juglans regia*]	*Xa21*	**1.30**	−3.54
isotig36058	RLK	putative receptor-like protein kinase At3g47110 [*Populus trichocarpa*]	*RLK*	**0.14**	−2.02
unigene37276	NLR	PREDICTED: TMV resistance protein N-like [*Malus domestica*]	*N*	−23.22	–**0.01**
unigene49085	NLR	TMV resistance protein N-like [*Arachis hypogaea*]	*N*	0.89	**2.13**
isotig52629	NLR	TMV resistance protein N [*Vigna radiata var. radiata*]	*N*	2.13	**3.37**
unigene104666	NLR	disease resistance-like protein DSC1 [*Citrus clementina*]	*DSC1*	0.00	**4.23**
isotig43179	NLR	PREDICTED: TMV resistance protein N-like isoform X2 [*Eucalyptus grandis]*	*N*	1.58	**2.84**

*Higher differential expression levels (Log2 fold change), in comparison to control samples, are highlighted in bold. RLP, receptor-like protein; RLK, receptor-like kinase; NLR, nucleotide-binding/leucine-rich-repeat receptor; Sus, susceptible; Res, resistant*.

## Discussion

A significant reprogramming of gene expression was observed in *P. pinaster* plants after inoculation with the PWN. This observation is not surprising, and it is in accordance with previous studies on *P. pinaster* inoculated with PWN (Santos and Vasconcelos, 2012; Gaspar et al., 2017, 2020) where susceptible plants have been analyzed. However, by focusing on both resistant and susceptible interactions in *P. pinaster* plants, we show here that although part of the transcriptional response to PWN was shared between both resistant and susceptible groups, significant qualitative and quantitative differences exist in gene expression. Importantly, some of these differences were confirmed to translate into relevant functional outcomes.

Several possible mechanisms involved in PWN resistance in *P. pinaster* are here described. Some clear differences in *P. pinaster* resistant and susceptible transcriptional responses were visible at 72 hpi, highlighting the activation of different phytohormone pathways, contrasting expression of resistance genes, lignin biosynthesis and, possibly, different levels of synthesis of other secondary metabolites. The induction of JA or SA in resistant and susceptible plants, respectively, can be pivotal to determine if the plant defense response is effective against PWN.

### Activation of SA and JA Pathways

The synthesis and accumulation of SA is induced by EDS1 and its interacting proteins, PAD4 and SAG101, which have also a role in repressing the JA pathway (Wiermer et al., 2005; Pieterse et al., 2011; Zhang and Li, 2019). Genes encoding for these proteins were more upregulated in susceptible plants, suggesting an activation of SA pathway at 72 hpi. At the same time, *MKS1*, which encodes for a protein that can repress SA signaling (Andreasson et al., 2005), was more expressed in resistant plants. This indicates that the activation of SA immune response at the studied time point may be characteristic of a susceptible response. In fact, levels of SA were higher in susceptible plants compared to resistant and controls, supporting this hypothesis.

Salicylic acid and jasmonic acid immune responses are often antagonistic, with SA pathway being mostly associated with response to biotrophic pathogens and JA pathway with response to necrotrophic pathogens and herbivory (Dar et al., 2015). Although JA and JA-Ile levels were similar in resistant and susceptible plants, SA may still inhibit JA response in susceptible plants, independently of JA biosynthesis (Caarls et al., 2015). The repression of JA pathway in susceptible plants, or activation in resistant plants, is supported by a higher expression of JA responsive genes in the latter. These include *chitinase, PR-4, PR-5, JAZ/Tify* transcription factors, and JA biosynthesis genes (Wasternack and Hause, 2013). Therefore, SA/JA antagonism may play a role in the outcome to PWN inoculation in *P. pinaster*, with the activation of SA pathway in susceptible plants and JA pathway in resistant ones, at 72hpi. In a recent study where hormone levels were analyzed for another *P. pinaster* family described in Carrasquinho et al. (2018), high levels of SA and jasmonic acid methyl ester (JA-ME) were detected in susceptible plants at 72 hpi (Rodrigues et al., 2021), supporting an important role for SA in PWN susceptibility. In *P. thurnbergii*, the induction of JA and SA responsive genes was also observed in susceptible plants (Hirao et al., 2012). Therefore, this hormonal response seems to be shared not only among *P. pinaster* families, but also susceptible pine species.

The role of ABA in plant immunity seems to be an ambivalent one (Ton et al., 2009). In some interactions, ABA can inhibit SA and JA/ET response (Lorenzo et al., 2004; Adie et al., 2007; Nahar et al., 2012; Hillwig et al., 2016), while in others it enhances JA response against fungi or herbivory (Ton and Mauch-Mani, 2004; Bodenhausen and Reymond, 2007; Ton et al., 2009; Liu et al., 2020b), activating the MYC branch of JA pathway (Pieterse et al., 2011; Vos et al., 2019). In this work, the similar levels of ABA seen in both inoculated and control plants suggests it does not play a part in defense response to PWN, as it was concluded for *P. pinaster* response to *Fusarium circinatum* (Hernandez-Escribano et al., 2020). However, the overexpression of both a positive regulator of ABA response, *PYL4*, and a repressor of ABA signaling, *PP2C* (Lackman et al., 2011), in resistant plants seems to indicate some role for the ABA pathway in the early stages of the infection. PYL4 is a receptor that recognizes ABA, activating ABA signaling pathway, and has been implicated in the crosstalk between ABA and JA during stress response (Lackman et al., 2011). Furthermore, PYL4 induces the expression of both ABA signaling pathway genes, such as *PP2C*, and JA signaling pathway genes, such as *MYC2* or *JAZ* TFs (Liu et al., 2020a). In this way, the upregulation of *PYL4* may lead to the activation of ABA pathway independently of ABA accumulation.

### Involvement of Pathogenesis-Related and Resistance Genes

The expression of *PR* and *chitinase* genes is commonly induced by defense response phytohormones (Van Loon et al., 2006; Pieterse et al., 2011). In this work, a higher expression of several chitin-binding PR-4 and chitinase encoding genes was observed in resistant plants. As chitin is a component of nematode eggshell (Fukushige and Futai, 1985; Holbein et al., 2016), these chitinases may compromise egg integrity and embryo development. In the RKN *Meloidogyne hapla*, treatments with chitinase plant extracts caused premature egg hatching and increased juvenile mortality (Mercer et al., 1992). It would be interesting to see if chitinase extracts from resistant *P. pinaster* plants have similar effects in PWM.

Several DEGs were identified as putative resistance genes. Interestingly, for many of these, different patterns of expression were detected in resistant and susceptible plants, emphasizing the differences between resistant and susceptible immune responses. For instance, the upregulation of a *FLS2* and a *RLP30* only in susceptible plants seem to reflect the activation of the SA pathway in these plants, since these genes have been described as SA responsive (Zhang and Li, 2019). On the other hand, the higher expression of NLR receptors in resistant plants may lead to the recognition of PWN effectors. Several studies have previously shown relevant roles for NRL receptors in plant resistance to parasitic nematodes (Sato et al., 2019; Zheng et al., 2021) and herbivorous insects (Hogenhout and Bos, 2011; Erb and Reymond, 2019). For instance, the NLR receptor encoded by gene *Mi-1.2* confers resistance to tomato root-knot nematodes (*Meloidogyne* spp.) (Milligan et al., 1998), the potato aphid (*Macrosiphum euphorbiae*) (Rossi et al., 1998), the white fly (*Bemisia tabaci*) (Nombela et al., 2003), and the tomato psyllid (*Bactericerca cockerelli*) (Casteel et al., 2006). In the same way, it is plausible that PWN delivers effectors to the plant cell cytoplasm while feeding through the stylet. The recognition of these effectors by NLR receptors could induce a stronger defense response in resistant plants.

### Induction of Secondary Metabolism Pathways

Secondary metabolites can be induced both by SA or JA, and their importance in plant defense response is well-established (Dar et al., 2015), particularly in conifer trees (e.g., Martin et al., 2002; Zhao et al., 2004; Zeneli et al., 2006; Moreira et al., 2009; Zulak et al., 2009; Zas et al., 2014). The overexpression of genes involved in the synthesis of secondary metabolites was induced by PWN inoculation in several pine species (Shin et al., 2009; Xu et al., 2013; Gaspar et al., 2017, 2020), particularly in resistant varieties (Kuroda et al., 2011; Hirao et al., 2012; Liu et al., 2017). In this work, similar results were obtained, with the induction of several genes involved in the phenylpropanoid biosynthesis pathway, including flavonoid or lignin biosynthesis, and the induction of a few genes involved in terpenoid biosynthesis.

The synthesis of terpene compounds has been implicated in resistance to several pests in pine species (Keeling and Bohlmann, 2006) and seems to be induced by JAs, including in *P. pinaster* (Moreira et al., 2009; Sampedro et al., 2011; Zas et al., 2014). For instance, specific diterpenes produced by AS and LPS, encoded by two genes that were here more expressed in *P. pinaster* resistant plants after PWN inoculation, were associated with *Pinus resinosa* resistance to bark beetle (Mason et al., 2017). An increased expression of terpene synthase genes was also observed in resistant *P. massoniana* plants in response to PWN (Liu et al., 2017), and the products of two of these enzymes, α-pinene and longifolene, directly inhibited the survival rate of PWN *in vitro* (Liu et al., 2020a). Other terpenoid compounds found in the resistant species *Pinus strobus* and *Pinus palustris* had nematicidal or repelling effect on PWN (Suga et al., 1993). In *P. pinaster*, plants can be grouped into several chemotypes according to the constitutive content in terpenoid compounds (Rodrigues et al., 2017; Gonçalves et al., 2020). Furthermore, feeding by the PWN insect vector *M. galloprovincialis* induced an increased production of these compounds in different patterns for the studied chemotypes, including α-pinene and longifolene (Gonçalves et al., 2020). The impact of these chemotypes on PWN resistance is, however, unknown and it would be relevant to investigate it. As synthesis of terpenes seems to be an effective and conserved response to herbivory, and more precisely to PWN, in several conifer species, distinct levels of specific compounds may have a significant impact on nematode survival in resistant *P. pinaster* plants.

Higher induction of the flavonoid biosynthesis pathway has consistently been found in nematode resistant varieties of several plants species (Chin et al., 2018). For instance, chalcone synthase (CHS) encoding genes were frequently more expressed in resistant plants (Chin et al., 2018) and the product of these enzymes, naringenin, caused reduced egg hatching in the burrowing nematode (Wuyts et al., 2006). In turn, LDOX is involved in the synthesis of another compound with a similar effect, kaempferol, and quercetin, which repelled root-knot nematode and burrowing nematode juveniles (Wuyts et al., 2006). As these flavonoids can affect nematode egg development, nematode mobility, and survival (Chin et al., 2018), the higher expression of genes encoding for CHS and LDOX enzymes in resistant *P. pinaster* plants may impact PWN and contribute to the observed phenotype. In *P. densiflora*, a higher expression of flavonoid biosynthesis pathway genes was also observed in resistant varieties (Kuroda et al., 2011).

The phenylpropanoid pathway was the secondary metabolism pathway more highly induced by PWN inoculation in *P. pinaster*, with special emphasis in the lignin biosynthesis pathway. Several genes specific to lignin synthesis, such as *PER* and *LAC* genes, had high expression levels in resistant plants and we were able to show that the higher gene expression translated into a significant increase in cell wall lignin content. The upregulation of *PER* genes and genes involved in cell wall strengthening was also associated with resistance in *P. thunbergii* (Hirao et al., 2012) and *P. massoniana* (Liu et al., 2017). Furthermore, higher lignification in regions surrounding plant tissue damaged by PWN has been observed in resistant *P. thunbergii* plants and associated with limited PWN migration (Kusumoto et al., 2014). Our results support that lignification seems to be an efficient strategy to reduce the spread of PWN, consequent plant tissue damage and likely to interfere with nematode feeding on plant cells (Naoumkina et al., 2010; Holbein et al., 2016).

In our data, it was possible to detect PWN gene expression during the infection process important for the successful infestation of plant tissues. Among these, were genes encoding for antioxidant proteins, which protect PWN from ROS produced by the plant during defense response (Espada et al., 2016). Resistance to oxidative stress has been positively correlated with PWN virulence (Vicente et al., 2015), suggesting that detoxification is essential for successful infection. As above mentioned, the expression of genes encoding the hydrogen peroxide producing enzymes GLOX, possibly involved in increased lignification, was higher in resistant plants. The production of higher amounts of this toxic compound in resistant plants may surpass the PWN capacity for detoxification and negatively influence the nematode performance. In inoculated *P. massoniana* plants, levels of hydrogen peroxide were slightly higher in resistant plants at 24 hpi (Liu et al., 2017), but no data was collected at 72 hpi. At 15 dpi, levels were reversed, being significantly lower in resistant plants, which was associated with a higher expression of oxidative stress response genes (Liu et al., 2017). In *P. pinaster*, as well as in *P. thunbergii* (Hirao et al., 2012), oxidative stress response genes were also more expressed in resistant plants, indicating that a better protection from oxidative damage is important for PWN resistance in several *Pinus* ssp.

## Concluding Remarks

Investigation of *P. pinaster* defense response to PWN inoculation in resistant plants has not been previously reported. Combining differential gene expression analysis with hormone and lignin quantification, we identified pathways and mechanisms potentially involved in PWN resistance. The induction of different hormone pathways, namely the SA pathway in susceptible plants vs. the JA pathway in resistant plants, and the higher lignification of plant tissues around the inoculation zone in resistant plants seem to be of great relevance for the phenotypic outcome after inoculation. Secondary metabolism pathways, resistance genes, and oxidative stress response genes also seem to play an important role in PWN resistance. The high expression of these groups of genes in resistant plants may interfere with nematode feeding, survival, mobility, and reproduction. This study provides the foundation to understand PWN resistance in *P. pinaster*, highlighting a set of candidate genes greatly relevant for future functional characterization studies. The use of compounds here associated with resistance, such as JA and secondary metabolites, for pest-management strategies has been previously suggested (e.g., Erbilgin et al., 2006; Goławska et al., 2014; Zas et al., 2014; Scalerandi et al., 2018) and should be explored.

Overall, the implication of several distinct pathways in the resistance of *P. pinaster* to PWN is in accordance with the quantitative nature of the resistance trait and the observation of intermediate symptoms from susceptibility to complete resistance in this study and in previous reports (Menéndez-Gutiérrez et al., 2017a; Carrasquinho et al., 2018). The search for genetic variation in the candidate genes here identified using high-throughput genotyping technologies, further supported by their possible co-location with quantitative trait loci (QTLs) currently under investigation in a larger number of families, may provide relevant molecular markers for identification of resistant genotypes. These approaches will greatly aid selection of individuals from the most resistant families to be used in current breeding or vegetative propagation programs.

## Data Availability Statement

The original contributions presented in the study are publicly available. The sequence data for this study has been submitted to the European Nucleotide Archive (ENA) at EMBL EBI under accession number PRJEB26836 (https://www.ebi.ac.uk/ena/browser/view/PRJEB26836).

## Author Contributions

IM, IC, and CM designed the experiment and performed the inoculation assay. IM performed the RNA experiments. VA and AG-C performed the hormone quantification experiment. IM performed data analysis, guided and supervised by LS and YV. IM and CM interpreted data and prepared the manuscript. All authors discussed the results and reviewed the manuscript.

## Conflict of Interest

The authors declare that the research was conducted in the absence of any commercial or financial relationships that could be construed as a potential conflict of interest.

## Supplementary Material

Supplementary Figure 1 Boxplots of the height and diameter at the base of the stem of inoculated plants (half-sib family 440) and *t*-test results for the comparison of these parameters' means between resistant (res) and susceptible (sus) plants. **(A)** Boxplot of height (cm) measurements. **(B)** Boxplot of diameter at the base of the stem (mm) measurements. Both measurements were made before inoculations. **(C)** *t*-Test results for heights comparison. **(D)** *t*-Test results for diameter comparison. *N*, number of samples. *SD*, standard deviation.

Supplementary Figure 2 Heatmaps representing the expression patterns of genes involved in secondary metabolism. **(A)** Flavonoid biosynthesis pathway. **(B)** Terpenoid biosynthesis pathways, including terpenoid backbone biosynthesis (Terp. Backbone), monoterpenoid biosynthesis, sesquiterpenoid biosynthesis, and diterpenoid biosynthesis pathways. The color gradient represents mean expression levels (logTPM) of each gene for control (C), susceptible (S), and resistant (R) samples.

Supplementary Figure 3 Heatmaps representing the expression patterns of genes involved in the synthesis of hydrogen peroxide **(A)** and response to oxidative stress **(B)**. The color gradient represents mean expression levels (logTPM) of each gene for control (C), susceptible (S), and resistant (R) samples.

Supplementary Figure 4 Heatmaps representing the expression patterns of hormone responsive transcription factors (TFs). Jasmonate responsive TFs JAZ/Tify **(A)** and ERF **(B)**, salicylic acid responsive TFs WRKY **(C)**, and abscisic acid responsive TFs NAC **(D)**. The color gradient represents mean expression levels (logTPM) of each gene for control (C), susceptible (S), and resistant (R) samples.

Supplementary Table 1 *De novo* assembly and *P. pinaster* reference transcriptome statistics.

Supplementary Table 2 Genes selected for quantitative RT-qPCR, respective primer sequences, amplicon size, and annealing temperatures used.

Supplementary Table 3 Genes expressed by *Bursaphelenchus xylophilus* in inoculated samples.

Supplementary Table 4 Differential expressed genes in susceptible plants when compared to controls (log2 fold change ≥ |2|, FDR corrected *p*-value (padj) ≤ 0.05). InterPro, KEGG, and blastx annotations are presented.

Supplementary Table 5 Differential expressed genes in resistant plants when compared to controls (log2 fold change ≥ |2|, FDR corrected *p*-value (padj) ≤ 0.05). InterPro, KEGG, and blastx annotations are presented.

Supplementary Table 6 GO terms enriched in the upregulated genes in susceptible samples when compared with controls, after trimming for redundancy.

Supplementary Table 7 GO terms enriched in the upregulated genes in resistant samples when compared with controls, after trimming for redundancy.

Supplementary Table 8 Complete list of genes annotated with DRAGO 2 tool.

Supplementary Table 9 Genes used for calculating average log2(fold change) expression levels for Figures 5, 6.

Data Sheet 1 Fasta file with *de novo* assembled *Pinus pinaster* transcripts.
