# Supplementary material for: Insights Into the Mechanisms Implicated in Pinus pinaster Resistance to Pinewood Nematode
Source: Front Plant Sci. 2021 Jun 10;12:690857. doi: 10.3389/fpls.2021.690857 (PMC8222992; doi:10.3389/fpls.2021.690857)
Supplement: Supplementary Figure 1 — Boxplots of the height and diameter at the base of the stem of inoculated plants (half-sib family 440) and t-test results for the comparison of these parameters' means between resistant (res) and susceptible (sus) plants. (A) Boxplot of height (cm) measurements. (B) Boxplot of diameter at the base of the stem (mm) measurements. Both measurements were made before inoculations. (C) t-Test results for heights comparison. (D) t-Test results for diameter comparison. N, number of samples. SD, standard deviation. [file Image_1.PDF]

a)

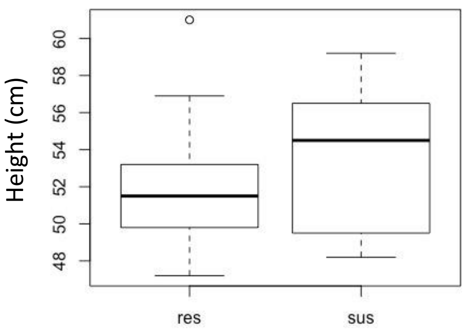

b)

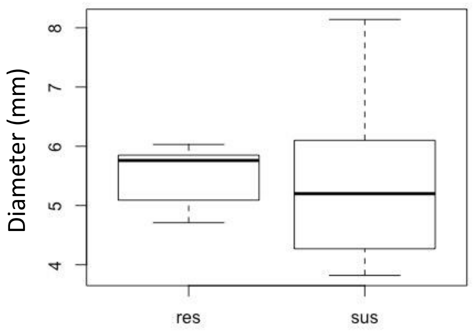

c)

	N	**Mean (cm)**	***SD***	***t***	***p*-value**
Res	7	52.3	4.0	-0.47	0.64
Sus	11	53.2	3.8		

d)

	N	**Mean (mm)**	***SD***	***t***	***p*-value**
Res	7	5.6	0.4	0.49	0.63
Sus	11	5.3	1.3		

Figure S1. Boxplots of the height and diameter at the base of the stem of inoculated plants (half-sib family 440) and *t*-test results for the comparison of these parameters' means between resistant (res) and susceptible (sus) plants. (a) Boxplot of height (cm) measurements. (b) boxplot of diameter at the base of the stem (mm) measurements. Both measurements were made before inoculations. (c) *t*-test results for heights comparison. (d) *t*-test results for diameter comparison. *N*, number of samples. *SD*, standard deviation.
