# Supplementary material for: Insights Into the Mechanisms Implicated in Pinus pinaster Resistance to Pinewood Nematode
Source: Front Plant Sci. 2021 Jun 10;12:690857. doi: 10.3389/fpls.2021.690857 (PMC8222992; doi:10.3389/fpls.2021.690857)
Supplement: Supplementary Figure 2 — Heatmaps representing the expression patterns of genes involved in secondary metabolism. (A) Flavonoid biosynthesis pathway. (B) Terpenoid biosynthesis pathways, including terpenoid backbone biosynthesis (Terp. Backbone), monoterpenoid biosynthesis, sesquiterpenoid biosynthesis, and diterpenoid biosynthesis pathways. The color gradient represents mean expression levels (logTPM) of each gene for control (C), susceptible (S), and resistant (R) samples. [file Image_2.PDF]

a)

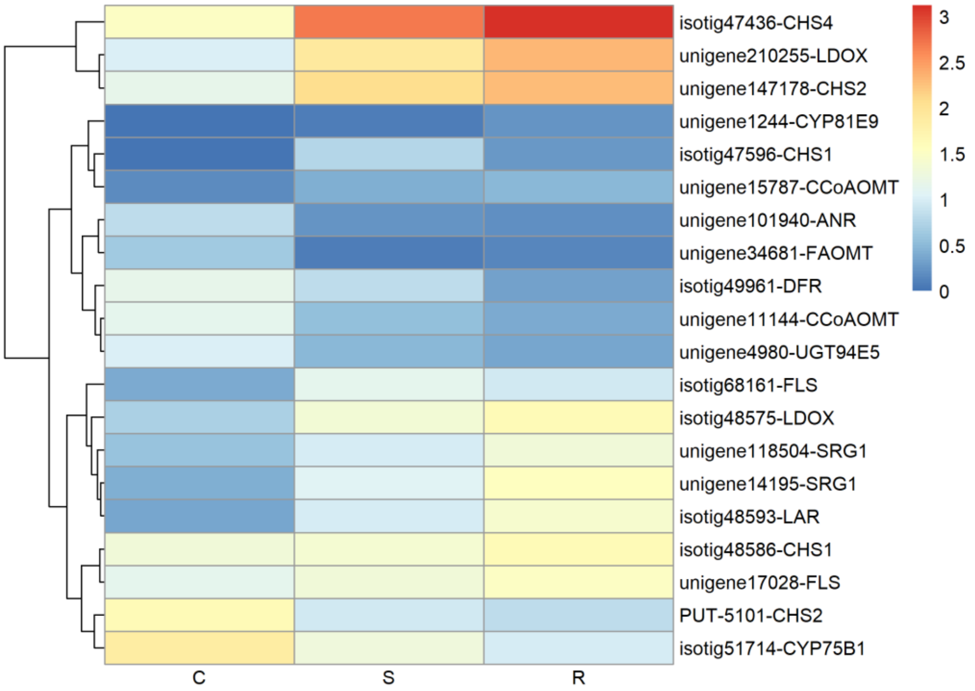

b)

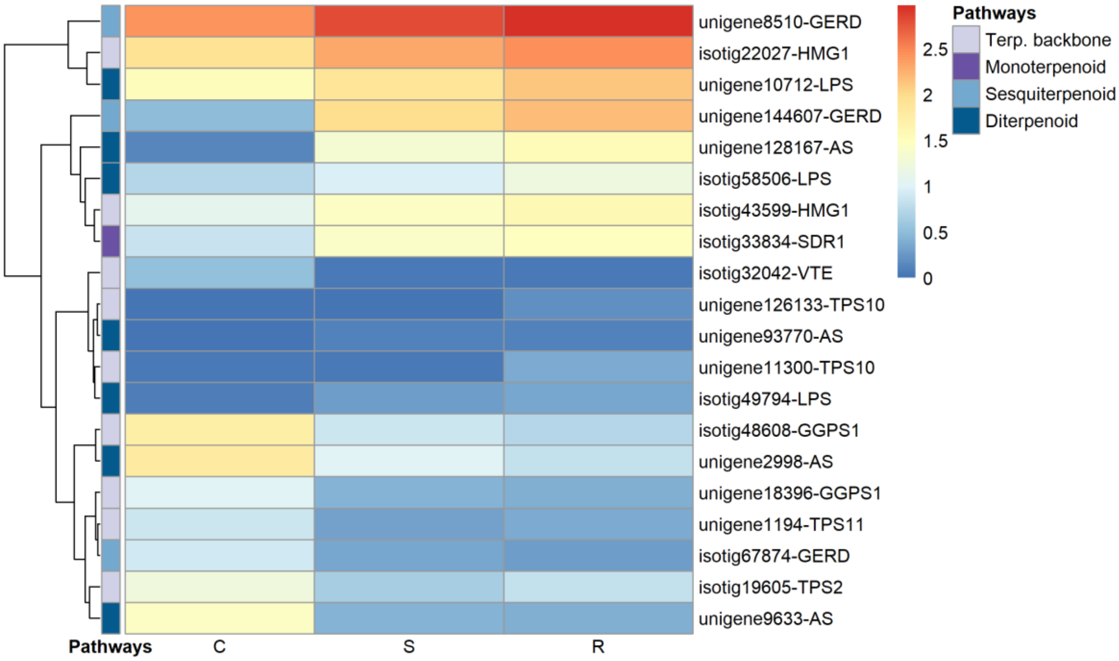

Figure S2. Heatmaps representing the expression patterns of genes involved in secondary metabolism. (a) Flavonoid biosynthesis pathway. (b) Terpenoid biosynthesis pathways, including terpenoid backbone biosynthesis (Terp. Backbone), monoterpenoid biosynthesis, sesquiterpenoid biosynthesis and diterpenoid biosynthesis pathways. The colour gradient represents mean expression levels (logTPM) of each gene for control (C), susceptible (S) and resistant (R) samples.
