# Supplementary material for: Insights Into the Mechanisms Implicated in Pinus pinaster Resistance to Pinewood Nematode
Source: Front Plant Sci. 2021 Jun 10;12:690857. doi: 10.3389/fpls.2021.690857 (PMC8222992; doi:10.3389/fpls.2021.690857)
Supplement: Supplementary Figure 3 — Heatmaps representing the expression patterns of genes involved in the synthesis of hydrogen peroxide (A) and response to oxidative stress (B). The color gradient represents mean expression levels (logTPM) of each gene for control (C), susceptible (S), and resistant (R) samples. [file Image_3.PDF]

a)

b)

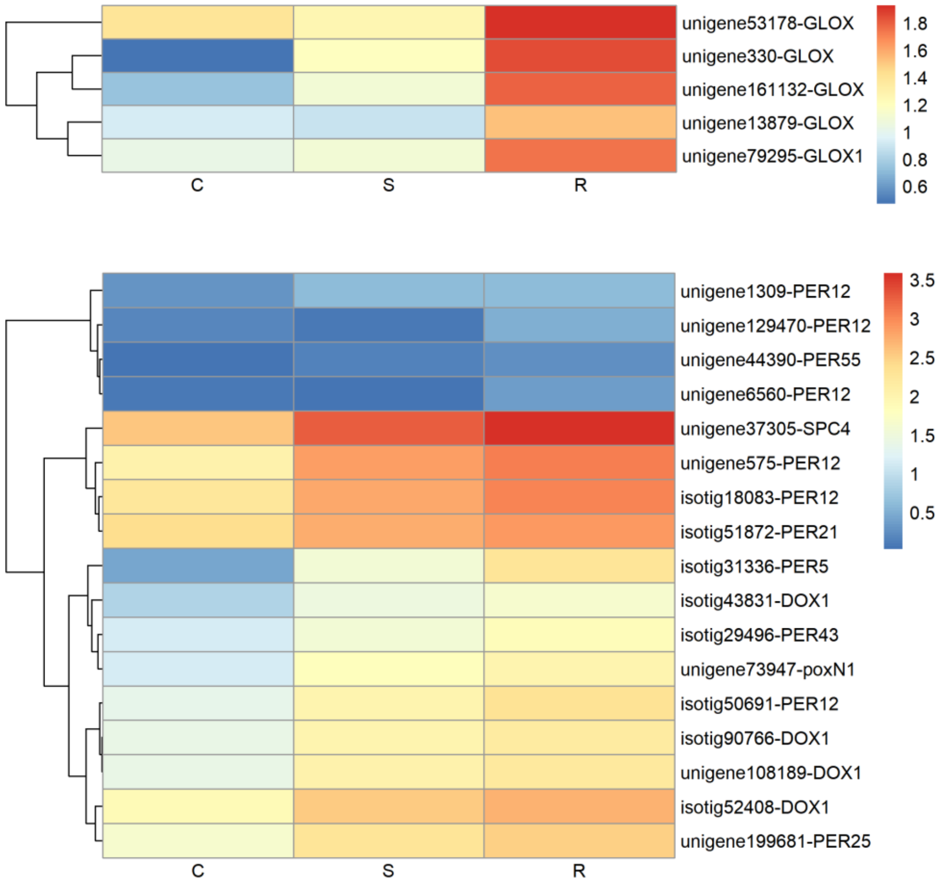

Figure S3. Heatmaps representing the expression patterns of genes involved in the synthesis of hydrogen peroxide (a) and response to oxidative stress (b). The colour gradient represents mean expression levels (logTPM) of each gene for control (C), susceptible (S) and resistant (R) samples.
