# Supplementary material for: Insights Into the Mechanisms Implicated in Pinus pinaster Resistance to Pinewood Nematode
Source: Front Plant Sci. 2021 Jun 10;12:690857. doi: 10.3389/fpls.2021.690857 (PMC8222992; doi:10.3389/fpls.2021.690857)
Supplement: Supplementary Figure 4 — Heatmaps representing the expression patterns of hormone responsive transcription factors (TFs). Jasmonate responsive TFs JAZ/Tify (A) and ERF (B), salicylic acid responsive TFs WRKY (C), and abscisic acid responsive TFs NAC (D). The color gradient represents mean expression levels (logTPM) of each gene for control (C), susceptible (S), and resistant (R) samples. [file Image_4.PDF]

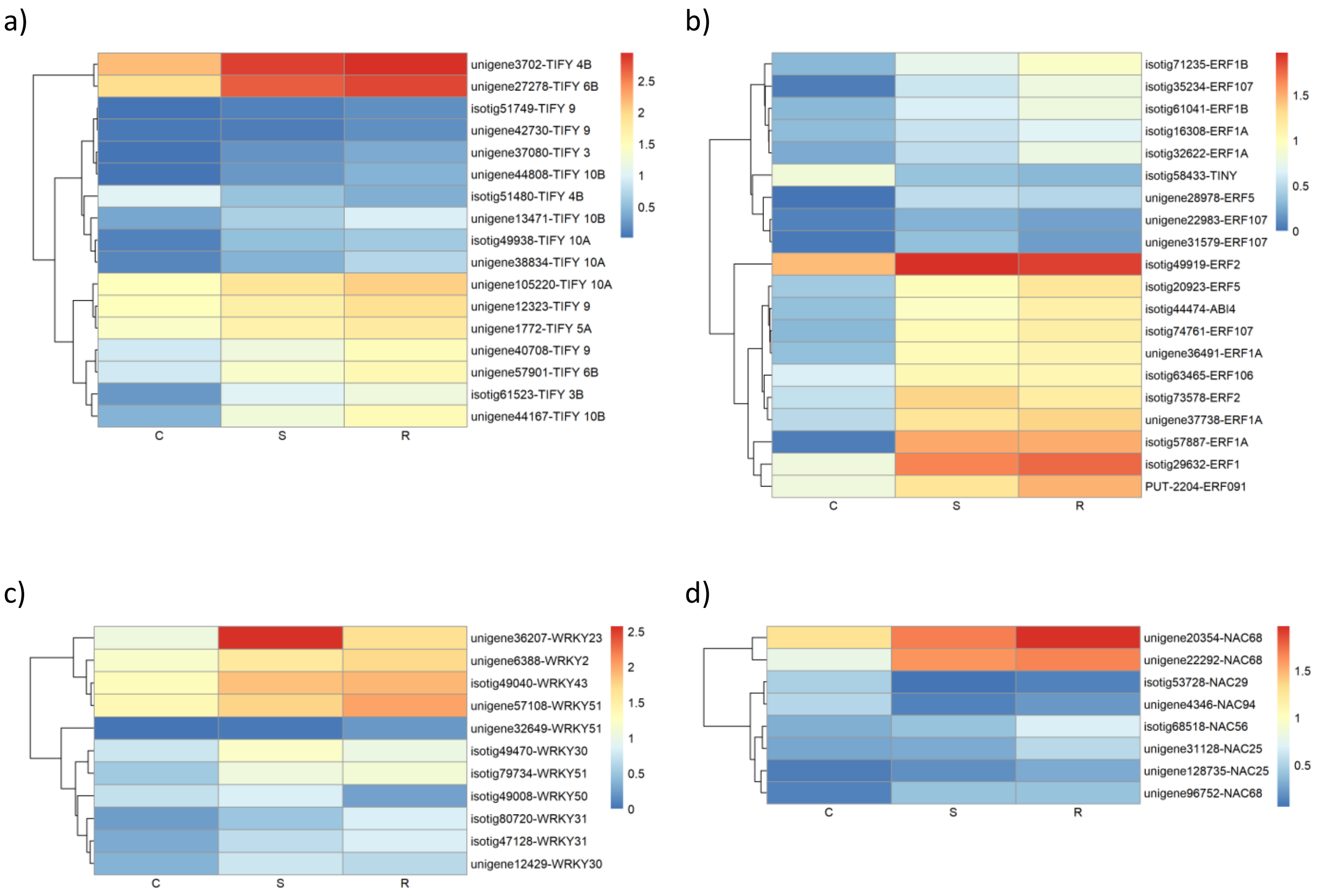

Figure S4. Heatmaps representing the expression patterns of hormone responsive transcription factors (TFs). Jasmonate responsive TFs JAZ/Tify (a) and ERF (b), salicylic acid responsive TFs WRKY (c) and abscisic acid responsive TFs NAC (d). The colour gradient represents mean expression levels (logTPM) of each gene for control (C), susceptible (S) and resistant (R) samples.
